# Transient Expression of Secretory IgA *In Planta* is Optimal Using a Multi-Gene Vector and may be Further Enhanced by Improving Joining Chain Incorporation

**DOI:** 10.3389/fpls.2015.01200

**Published:** 2016-01-11

**Authors:** Lotte B. Westerhof, Ruud H. P. Wilbers, Debbie R. van Raaij, Christina Z. van Wijk, Aska Goverse, Jaap Bakker, Arjen Schots

**Affiliations:** Laboratory of Nematology, Plant Science Group, Wageningen UniversityWageningen, Netherlands

**Keywords:** secretory IgA, plant-based expression, Ustekinumab, *N*-glycosylation, heteromultimeric, protein complex assembly, co-infiltration, multi-gene vector

## Abstract

Secretory IgA (sIgA) is a crucial antibody in host defense at mucosal surfaces. It is a promising antibody isotype in a variety of therapeutic settings such as passive vaccination and treatment of inflammatory disorders. However, heterologous production of this heteromultimeric protein complex is still suboptimal. The challenge is the coordinate expression of the four required polypeptides; the alpha heavy chain, the light chain, the joining chain, and part of the polymeric-Ig-receptor called the secretory component, in a 4:4:1:1 ratio. We evaluated the transient expression of three sIgAκ variants, harboring the heavy chain isotype α1, α2m1, or α2m2, of the clinical antibody Ustekinumab *in planta*. Ustekinumab is directed against the p40 subunit that is shared by the pro-inflammatory cytokines interleukin (IL)-12 and IL-23. A sIgA variant of this antibody may enable localized treatment of inflammatory bowel disease. Of the three different sIgA variants we obtained the highest yield with sIgA1κ reaching up to 373 μg sIgA/mg total soluble protein. The use of a multi-cassette vector containing all four expression cassettes was most efficient. However, not the expression strategy, but the incorporation of the joining chain turned out to be the limiting step for sIgA production. Our data demonstrate that transient expression *in planta* is suitable for the economic production of heteromultimeric protein complexes such as sIgA.

## Introduction

Secretory IgA (sIgA) is the predominant antibody type in mucosal secretions of the human body and plays a key role in the first line of defense against mucosal pathogens. While human serum IgA is primarily monomeric, B cells in mucosa-associated lymphoid tissues secrete IgA in a dimeric form through incorporation of the joining chain. Dimeric (d)IgA can bind the polymeric immunoglobulin receptor on the basolateral surface of epithelial cells where after the protein complex is transcytosed to the luminal side of the cell. Here the receptor is cleaved and a part of the receptor called the secretory component stays associated with the protein complex that is now referred to as secretory (s)IgA. Both dIgA and sIgA have immunological roles without development of inflammation. Mucosal antigens and/or pathogens can be bound by d/sIgA just before, during (e.g., intracellular pathogens in the epithelial cells) or after transcytosis and are thereby excluded from the mucosal tissue. On the luminal side of epithelial cells glycans on the secretory component facilitate binding to the mucus thereby enabling clearance of sIgA-antigen/pathogen complexes. This concept of antigen/pathogen binding and clearance that does not lead to inflammation is referred to as immune exclusion and is believed not only to play a role in combating mucosal pathogens, but also in controlling commensal bacteria. Inflammation does not develop because there is limited presence of FcαRI positive immune cells in the mucosal area and sIgA has reduced affinity for FcαRI. In order to fulfill these roles the human body secretes 40–60 mg sIgA per kg body weight each day (Conley and Delacroix, 1987).

Two isotypes of IgA exist, IgA1 and IgA2 of which the latter occurs in two allotypes, IgA2m1 and IgA2m2. All three alpha heavy chains consist of one variable domain, three constant domains and an extended tailpiece allowing IgA to dimerise by incorporation of the joining chain. There are three major differences between these IgA variants. First, IgA1 has an extended hinge region. This extension is heavily *O*-glycosylated, which is suspected to play a conformational role (Narimatsu et al., 2010), allowing the binding of more distantly spaced antigens. Second, alpha heavy chains of isotypes 1 and 2m2 are covalently linked to their light chains via a disulphide bridge. No such linkage exists in IgA2m1, which allows the formation of a intermolecular disulphide bridge between the two light chains. Third, all variants differ from each other in the number of *N*-glycosylation sites they carry (2, 4, and 5 for IgA1, IgA2m1 and IgA2m2, respectively). The ratio wherein sIgA isotypes are present depends on the mucosal area, which in turn is most likely the result of the presence of specific bacteria as the IgA1 hinge region is sensitive to bacterial proteases (Vaerman et al., 1968; Stoop et al., 1969; Delacroix et al., 1982; Kerr, 1990).

The use of recombinant sIgA in passive mucosal immunotherapy in humans and livestock has been suggested as a good alternative for antibiotics. Several *in vivo* studies demonstrated (s)IgA’s potential to locally control mucosal pathogens, such as *Mycobacterium tuberculosis* in the lungs (Williams et al., 2004), *Streptococcus mutans* in the mouth (Ma et al., 1998), and *Salmonella typhimurium*, *Vibrio cholera*, and *Cryptosporidium parvum* in the gastrointestinal tract (Enriquez and Riggs, 1998). When sIgA would be directed against pro-inflammatory cytokines and administered to the gut, it may relieve symptoms and induce remission in patients suffering from inflammatory bowel diseases (IBDs). Current treatment of IBD often includes systemic application of anti-TNF-α or anti-IL-12/23 antibodies. However, application of anti-TNF-α antibodies has been associated with the onset of tuberculosis (Keane et al., 2001). Non-systemic cytokine neutralization may reduce such infection risks and other side effects. Because sIgA is stable in the gut, sIgA-based therapy could be localized. Luminal administration of an anti-TNF-α antibody was effective in mouse models of IBD (Bhol et al., 2013) and local drug administration has been suggested to be important for efficacy of IBD therapies (Neurath, 2014).

Plants are a promising production platform for pharmaceutical proteins. As eukaryotes they are able to correctly fold complex proteins and assemble protein complexes such as virus like particles (Chen and Lai, 2013) and antibodies (De Muynck et al., 2010). Compared to mammalian cells, plants are a more economic production platform, as they do not require expensive cell culture conditions. Furthermore, the *N*-glycosylation pathway has been engineered to facilitate expression of antibodies with humanized *N*-glycans to allow effector functions (Bosch et al., 2013). Also, the engineering of mammalian mucin-type *O*-glycans has been achieved in plants (Castilho et al., 2012; Yang et al., 2012).

Plant-based expression of sIgA was achieved by stable transformation of the four individual genes required for sIgA assembly followed by crossing the highest producers (Ma et al., 1995; Paul et al., 2014). A drawback of this strategy is that it is a lengthy and laborious process. Transient expression is much faster and almost always results in higher yields, as there is no constraint on expression imposed by the site of insertion in the plant genome. To achieve transient expression of more than one gene simultaneously, *Agrobacterium* cultures harboring vectors for the expression of the individual genes need to be co-infiltrated or a multi-cassette vector facilitating expression of all genes should be used. The risk with co-infiltration is that cells may not be transformed with all genes, but use of a multi-cassette vector may be impractical if expression of the individual proteins needs to be adjusted to reflect the stoichiometry of the protein complex. Transient expression of chicken sIgA was achieved by co-infiltration (Wieland et al., 2006) and human sIgA was transiently expressed with a multi-cassette vector system (Juarez et al., 2013; Paul et al., 2014). While above-mentioned studies achieved sIgA expression, they also showed the presence of a large proportion of monomeric IgA as well as other assembly intermediates. The presence of these assembly intermediates complicates down-stream processing.

The objective of this study was to evaluate the plant-based expression and assembly of three sIgA variants of the clinical antibody Ustekinumab (CNTO1275) to unravel limitations in sIgA assembly. This antibody has specificity for the p40 subunit shared by interleukin-12 and interleukin-23 and may be used in IBD therapy. Changing the backbone from IgG to sIgA may enable local administration. First, we evaluated the transient expression of all individual genes required for sIgA assembly whereby the three alpha heavy chain types 1, 2m1, and 2m2 were included. Next we compared co-expression with the use of multi-cassette vectors. The use of a multi-cassette vector including all genes increased sIgA expression threefold and decreased the presence of the intermediate dIgA compared to co-infiltration. However, sIgA expression may be further optimized, because we conclude that inefficient incorporation of the joining chain limits sIgA assembly. This maybe a consequence of inefficient *N*-glycosylation of the IgA tailpiece and/or joining chain. Improved *N*-glycosylation may be the key to enhance sIgA assembly en boost yield even further.

## Experimental Procedures

### Construct Design

**FIGURE 1:**
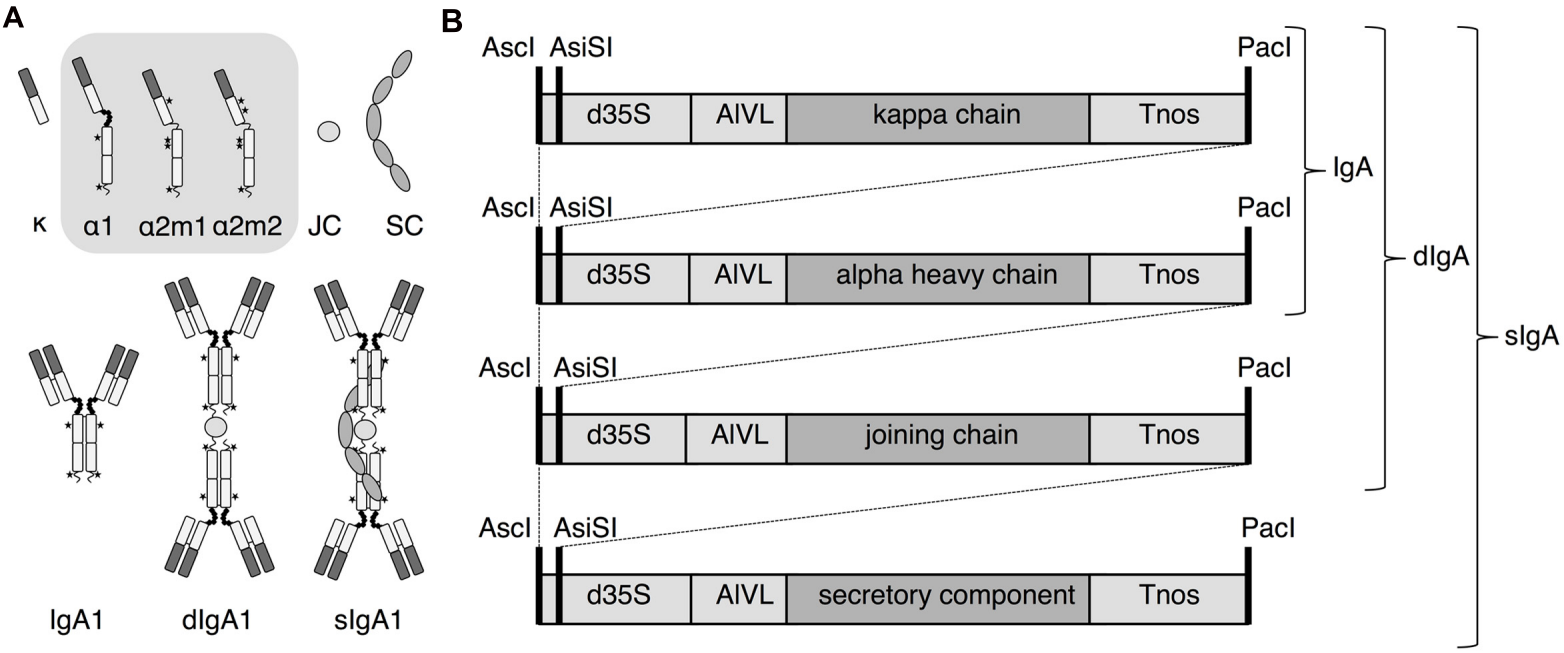
**Overview of sIgA assembly and expression. (A)** Individual proteins, alpha heavy chain 1 (α^1^), 2m1 (α^2m1^), or 2m2 (α^2m2^), kappa chain (κ), joining chain (JC), and secretory component (SC), and protein complexes IgA1κ, dimeric (d)IgA1κ and secretory (s)IgA1κ. **(B)** Expression cassettes with 35S promoter of the *Cauliflower mosaic virus* with duplicated enhancer (d35S), *Agrobacterium tumefaciens* nopaline synthase transcription terminator (Tnos), translational leader sequence of the Alfalfa mosaic virus RNA 4 (AlVL) and AscI, AsiSI and PacI restriction sites are indicated. Co-expression is required for IgA, dIgA, and sIgA assembly as indicated by the accolades.

GeneArt (Thermo Fisher, Bleiswijk, The Netherlands) synthesized all below-mentioned gene fragments except the constant region of human immunoglobulin alpha-2m1 (AAB59396.1), which was amplified from the human transcriptome library MegaMan (Agilent Technologies, Middelburg, The Netherlands). Before gene synthesis, undesired restriction sites were removed from the sequences of the constant domains of human immunoglobulin alpha-1 (AAC82528.1) and kappa (AAA59000.1) chains, joining chain (AK312014.1) and secretory component (codons 1–764 of the polymeric immunoglobulin receptor; AAB23176.1). To obtain the sequence for the constant domains of human immunoglobulin alpha-2m2 the sequence of human immunoglobulin alpha-2m1 was adapted (P93S, P102R, F279Y, D296E, L319M, V326I, and V335A) based on the amino acid sequence P01877 (Uniprot). The variable regions of the clinical antibody Ustekinumab (CNTO-1275) and the signal peptide of the *Arabidopsis thaliana* chitinase gene (AAM10081.1) were recoded from the amino acid sequence using codons preferred by *Nicotiana benthamiana*. For subsequent cloning and assembly of the alpha heavy chain genes, the gene fragments were flanked by the following restriction sites at the 5′- and 3′-end: NcoI-EagI, EagI-NheI, NheI-KpnI, for the signal peptide, the heavy chain variable and alpha heavy chain constant regions, respectively. For subsequent cloning and assembly of the kappa chain, the gene fragments were flanked by the following restriction sites at the 5′- and 3′-end: NcoI-EagI, EagI-BsiWI, BsiWI-KpnI for the signal peptide, the kappa chain variable and constant region, respectively. For subsequent cloning of the joining chain and secretory component the sequences were flanked by NcoI-KpnI at the 5′- and 3′-end, respectively. None of the restriction sites used introduced extra amino acids except NcoI, which in some cases introduced an extra alanine after the start methionine. Genes were ligated into the shuttle vector pRAPa, a pRAP (or pUCAP35S) derivative (van Engelen et al., 1994) modified to include an AsiSI restriction site by introduction of the self-annealed oligo 5′- AGCTGGCGATCGCC -3′ into a HindIII linearized pRAP. In pRAPa all open reading frames are placed under the control of the 35S promoter of the *Cauliflower mosaic virus* with duplicated enhancer (d35S) and the *Agrobacterium tumefaciens* nopaline synthase transcription terminator (Tnos). A 5′ leader sequence of the Alfalfa mosaic virus RNA 4 (AlMV) is also included between the promoter and gene to boost translation. From pRAPa the expression cassettes were digested with AscI and PacI, confirmed by sequencing, and ligated into the expression vector pHYG (Westerhof et al., 2012). Use of the restriction sites AscI and AsiSI allowed subsequent introduction of expression cassettes as AsiSI creates the same overhang as PacI (**Figure 1B**). Three multi-cassette vectors were generated; one that combined the alpha chain-1 and the light chain, one that combined the alpha chain-1, the light chain and the joining chain and one that combined the alpha chain-1, the light chain, the joining chain and the secretory component. Expression vectors were transformed to *Agrobacterium tumefaciens* strain MOG101 for transient plant expression.

### Transient Plant Transformation

*Agrobacterium* clones were cultured overnight (o/n) at 28°C in LB medium (10 g/l pepton 140, 5 g/l yeast extract, 10 g/l NaCl with pH 7.0) containing 50 μg/ml kanamycin and 20 μg/ml rifampicin. The optical density (OD) of the o/n cultures was measured at 600 nm and used to inoculate 50 ml of LB medium containing 200 μM acetosyringone and 50 μg/ml kanamycin with *x* μl of culture using the following formula: *x* = 80000/(1028^∗^OD). OD was measured again after 16 h and the bacterial cultures were centrifuged for 15 min at 2800 × *g*. The bacteria were resuspended in MMA infiltration medium (20 g/l sucrose, 5 g/l MS-salts, 1.95 g/l MES, pH 5.6) containing 200 μM acetosyringone. For co-expression of genes *Agrobacterium* cultures harboring expression vectors for individual gene expression were mixed prior to infiltration or a multi-cassette vector was used. The final OD of each *Agrobacterium* culture in an infiltration mix was 0.5 unless indicated otherwise. The total OD of the infiltration mix was kept the same within an experiment by using an *Agrobacterium* culture harboring an empty vector if needed. The Tomato bushy stunt virus (TBSV) silencing inhibitor p19 was always co-expressed (Voinnet et al., 2003). After 1–2 h incubation of the infiltration mix at room temperature, the two youngest fully expanded leaves of 5–6 weeks old *Nicotiana benthamiana* plants were infiltrated completely. Infiltration was performed by injecting the *Agrobacterium* suspension into a *Nicotiana benthamiana* leaf at the abaxial side using a needleless 1 ml syringe. Infiltrated plants were maintained in a controlled greenhouse compartment (UNIFARM, Wageningen) and infiltrated leaves were harvested at selected time points.

### Total Soluble Protein Extraction

Leaf disks were taken from fully infiltrated leaves and immediately snap-frozen. Plant material was ground first in liquid nitrogen and then in 2 ml ice-cold extraction buffer [50 mM phosphate-buffered saline (PBS) pH = 7.4, 100 mM NaCl, 10 mM ethylenediaminetetraacetic acid (EDTA), 0.1% v/v Tween-20, 2% w/v immobilized polyvinylpolypyrrolidone (PVPP)] per g fresh weight using a TissueLyser II (Qiagen, Venlo, The Netherlands). Crude extract was clarified by centrifugation at 16.000 × *g* for 5 min at 4°C.

### IgA and sIgA Quantification

IgA and sIgA concentrations in crude extracts were determined by sandwich ELISA. ELISA plates (Greiner Bio One; Alphen aan den Rijn, The Netherlands) were coated o/n at 4°C in a moist environment with goat polyclonal anti-human kappa antibody (Sigma–Aldrich; Zwijndrecht, The Netherlands) in coating buffer (eBioscience, Vienna, Austria). After this and each following step the plate was washed five times with 30 s intervals in PBST (1x PBS, 0.05% Tween-20) using an automatic plate washer model 1575 (BioRad; Veenendaal, The Netherlands). The plate was blocked with assay diluent (eBioscience) for 1 h at room temperature. Samples and a standard line were loaded in serial dilutions and incubated for 1 h at room temperature. For IgA determination recombinant human IgA1κ (InvivoGen; Toulouse, France) was used as a standard in a twofold dilution series from 100 to 0.31 ng/ml in assay diluent. For sIgA determination colostrum purified sIgA (Sigma–Aldrich) was used as a standard in a twofold dilution series from 1000 to 3.1 ng/ml in assay diluent. Hereafter a HRP-conjugated goat polyclonal antibody directed against the constant domains of human IgA (Sigma–Aldrich) or a biotinylated goat polyclonal antibody directed against the human secretory component (Sigma–Aldrich) was used for detection of IgA and sIgA, respectively. Avidin-HRP conjugate (eBioscience) was used to bind the biotin of the anti-secretory component antibody. 3,3′,5,5′-Tetramethylbenzidine (TMB) substrate (eBioscience) was added and coloring reaction was stopped using 0.18 M sulphuric acid after 1–30 min. OD read outs were performed using the model 680 microplate reader (BioRad) at 450 nm with correction filter of 690 nm. For sample comparison the total soluble protein (TSP) concentration was determined using the BCA Protein Assay Kit (Pierce) according to supplier’s protocol using bovine serum albumin (BSA) as a standard.

### Protein Analysis by Western Blot

For western blot analysis clarified protein extracts were desalted using a Sephadex G25 (VWR International; Amsterdam, The Netherlands) column prior to BCA analysis. One microgram (unless otherwise indicated) of TSP was separated under reducing or non-reducing conditions by SDS-PAGE on in house made 6 or 12% Bis-Tris gels. Recombinant IgA1κ (InvivoGen) and/or colostrum purified sIgA (Sigma–Aldrich) or recombinant joining chain (Sino Biological; Cologne, Germany) were used as controls. Proteins were transferred to an Invitrolon^TM^ PVDF membrane (Invitrogen) by a wet blotting procedure (Life technologies; Bleiswijk, The Netherlands). Thereafter the membrane was blocked in PBST-BL (PBS containing 0.1% v/v Tween-20 and 5% w/v non-fat dry milk powder) for 1 h at room temperature, followed by overnight incubation with a goat anti-human kappa (Sigma–Aldrich), goat anti-human immunoglobulin alpha (Sigma–Aldrich), goat anti-joining chain (Nordic Immunological Laboratories; Tilburg, The Netherlands) or goat anti-secretory component (Sigma–Aldrich) antibody. The membrane was washed five times in PBST (PBS containing 0.1% v/v Tween-20). There after a HRP-conjugated anti-goat IgG antibody (Jackson ImmunoResearch; Suffolk, UK) was incubated with the membrane where after washing steps were repeated. The SuperSignal West Dura substrate (Thermo Fisher Scientific; Etten-Leur, The Netherlands) was used for visualization. Pictures were taken using a G:BOX Chemi System device (SynGene; Cambridge, UK).

## Results

### Expression of Individual Components Required for sIgA Assembly

**FIGURE 2:**
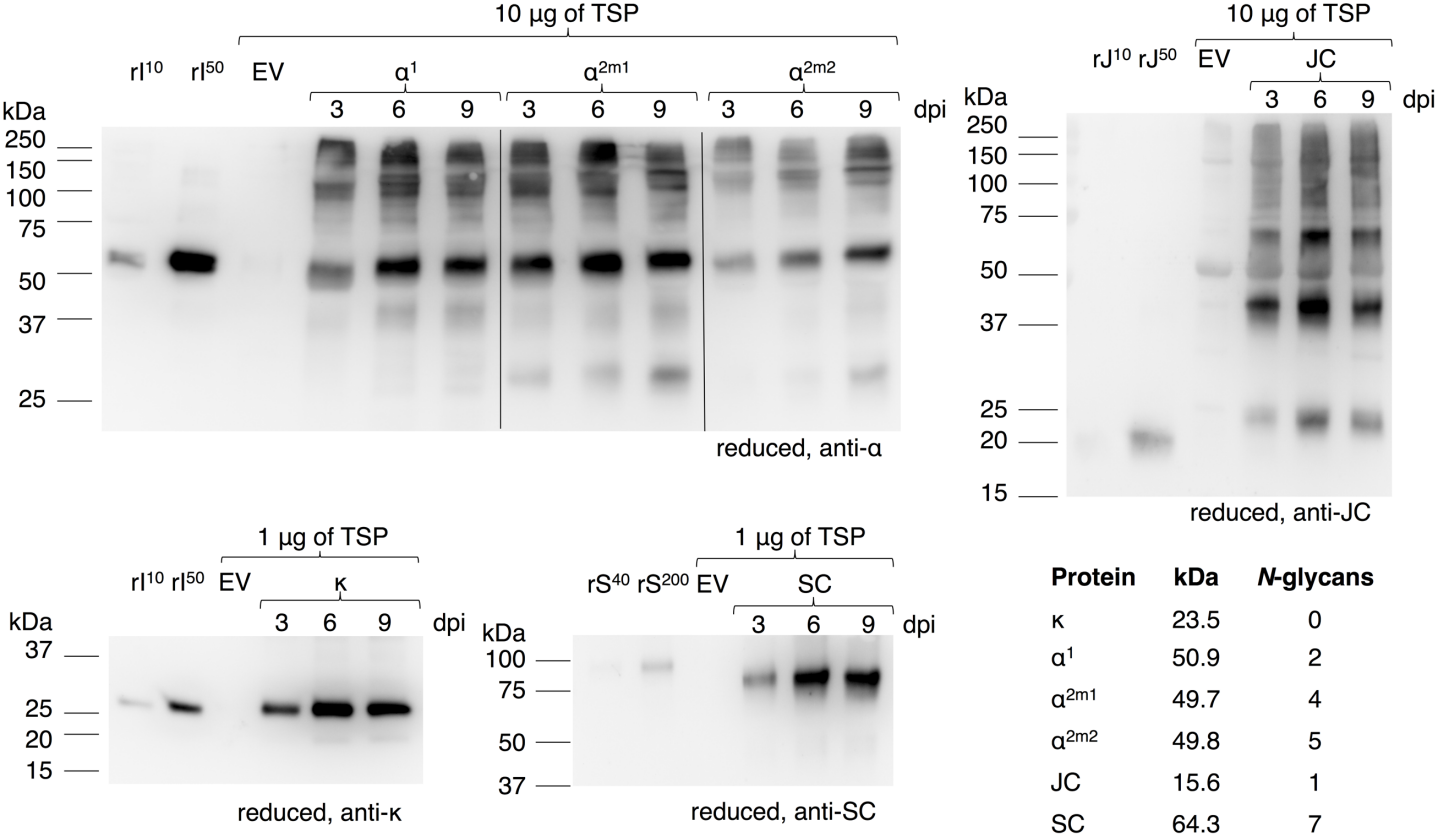
**Plant-based expression of individual genes required for sIgA assembly**. Leaves were agro-infiltrated for the expression of alpha heavy chain 1 (α^1^), 2m1 (α^2m1^), 2m2 (α^2m2^), kappa chain (κ), JC, or SC and harvested 3, 6, and 9 dpi. Total soluble protein (TSP) was extracted and separated by SDS-PAGE under reducing conditions followed by visualization on a western blot using protein specific antibodies as indicated. As controls 10 or 50 ng of recombinant IgA1κ (rI^10/50^), recombinant joining chain (rJ^10/50^) or 40 or 200 ng purified sIgA (rS^40/200^, containing ∼10 or 50 ng SC) and an empty vector (EV) sample were used. The table states the expected protein size as calculated by the amino acid sequences of the mature protein and the expected number of *N*-glycans according to literature.

In order to achieve *in vivo* assembly of a heteromultimeric protein complex all required genes should be expressed simultaneously in the same cell and preferably at the right stoichiometry. The sIgA protein complex comprises four proteins. An overview of the individual proteins and the protein complexes IgA, dimeric (d)IgA and sIgA based on alpha heavy chain isotype 1 is given in **Figure 1A**. Expression cassettes and the combinations thereof to achieve expression of these protein complexes are given in **Figure 1B**. To evaluate the level and course of expression of the individual genes we first expressed them individually and monitored expression over time [3, 6, and 9 days post infiltration (dpi)] using western blot analysis (**Figure 2**). For the alpha heavy chain the isotypes 1, 2m1, and 2m2 were evaluated. Upon expression of the alpha heavy chains and the kappa light chain bands were detected at the expected sizes that are assumed to represent the intact proteins. Furthermore, for all heavy chains several bands >100 kDa and a few faint bands <50 kDa were observed, which most likely represent multimers and products of proteolytic degradation, respectively. Upon expression of the secretory component a band was detected that migrates ∼10 kDa lower compared to the secretory component of the sIgA control. This may be explained by a difference in the number and/or type of *N*-glycans received by the secretory component when expressed in plants. The secretory component has seven confirmed glycosylation sites, but these may not (all) be glycosylated in plants. Furthermore, the most common *N*-glycans of plant-secreted proteins are 0.7–1.1 kDa smaller than most typical *N*-glycans found on human secretory component (Royle et al., 2003) which could already account for ∼10 kDa difference in protein size. Upon expression of the joining chain many bands were detected, but all migrate higher as expected for a single joining chain (15.6–17.1 kDa, depending on glycosylation) and most of them likely represent dimers/multimers. Also the *E. coli* produced recombinant joining chain (expected size ∼17 kDa) displays aberrant migration behavior and migrates around 20 kDa.

The course of expression of the individual proteins is similar and peaks at 6 dpi, except for alpha heavy chain 2m2, which peaks at 9 dpi. However, yields of the individual proteins vary. By comparing the band intensity of each individual protein to the recombinant controls we estimate yields between 1 and 5 μg/mg TSP for the alpha heavy chains, between 5 and 20 μg/mg TSP for the joining chain and between 50 and 200 μg/mg TSP for the kappa chain and the secretory component at dpi 6. Yield estimation of the heavy chains and joining chain is solely based on the intact monomeric proteins and is most likely underestimated due to the presence of multimers. Nonetheless, as the stoichiometric ratio between the heavy chain, the kappa chain, the joining chain and the secretory component is 4:4:1:1, we assume heavy chain expression to be the limiting factor for sIgA assembly if stabilization of individual proteins upon co-expression would not occur.

### A Multi-Cassette Vector is Most Efficient for Transient Expression of sIgA

**FIGURE 3:**
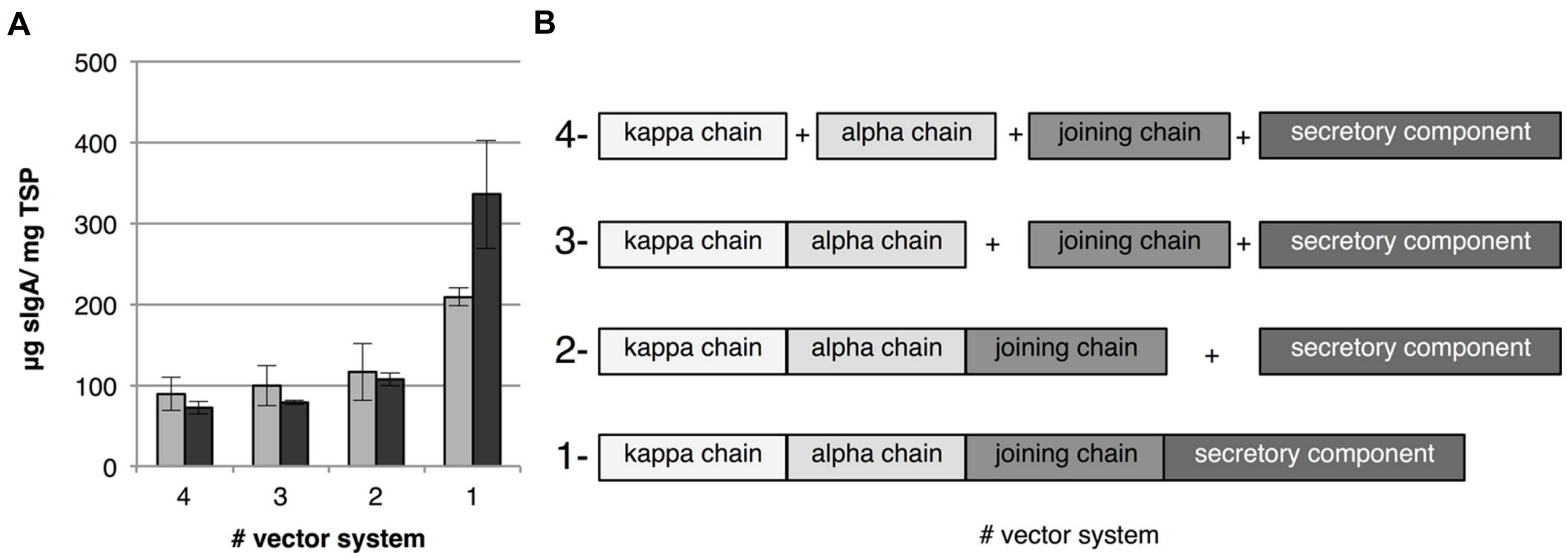
**sIgA yield upon transient transformation using co-infiltration and multi-cassette vectors. (A)** Average sIgA yield 6 dpi of three biological replicates in leaf extracts when co-infiltrating all genes individually, co-infiltrating the secretory component and joining chain with the multi-cassette vector for IgA1κ expression, co-infiltrating the secretory component and the multi-cassette vector for dIgA expression and the infiltration of the multi-cassette vector for sIgA expression indicated as the 4-, 3-, 2-, and 1-vector system, respectively. The final OD of the infiltration mix was set to 2.5 using either an *Agrobacterium* culture carrying an EV (light gray bars) or the OD of the *Agrobacterium* culture carrying the multi-cassette vector was increased (dark gray bars). Error bars indicate standard error. **(B)** A schematic overview of the expression cassettes in the 4-, 3-, 2-, and 1-vector systems.

Co-expression in transient transformation can be achieved in two ways, either by co-infiltration of *Agrobacterium* cultures harboring a vector for each individual gene or by using a multi-cassette vector facilitating expression of all genes. Which strategy would lead to the best co-ordinated expression is unclear. With co-infiltration it is possible that not all cells are transformed with all expression cassettes. The use of a multi-cassette vector would ensure that a transformed cell receives all genes, however, transformation may be less efficient due to the larger size of the T-DNA. Therefore we used both strategies and combinations thereof to express all genes needed for sIgA complex formation. We successfully constructed several multi-cassette vectors for expression of IgA1κ (alpha heavy chain 1 and kappa chain), dIgA1κ (alpha heavy chain 1, kappa chain and joining chain) and sIgA1κ (all four genes) whereby all genes are under control of the same promoter and terminator (**Figure 1B**). Subsequently, sIgA was expressed by co-infiltration of all genes individually (4-vector system), co-infiltration of the secretory component and the joining chain with the multi-cassette vector for IgA1κ expression (3-vector system), co-infiltration of the secretory component and the multi-cassette vector for dIgA1κ expression (2-vector system) and the infiltration of the multi-cassette vector for sIgA1κ expression (1-vector system; **Figure 3B**). **Figure 3A** shows the average sIgA yield of three biological replicates as determined by ELISA. To correct for the lower OD of the final *Agrobacterium* infiltration mix of the 3-, 2-, and 1-vector systems compared to the 4-vector system, an *Agrobacterium* culture carrying an empty vector (EV) was used to increase the OD (gray bars) or the concentration of the *Agrobacterium* carrying the multi-cassette vector was increased (dark gray bars). In both situations the yield is similar between the 4-, 3-, and 2-vector systems. Surprisingly, however, the use of the 1-vector system increases yield twofold to threefold. In the situation where the OD of the *Agrobacterium* cultures were supplemented with EV culture (gray bars), the twofold yield increase may be explained by an increased number of cells that receives all genes. In the case where we compensated the OD of the *Agrobacterium* cultures with cultures harboring the multi-cassette vector (dark gray bars) we were able to enhance sIgA yield 1.6-fold further. Use of a higher OD of an *Agrobacterium* culture often increases yield, as more T-DNA copies are transferred to the plant cell. Noteworthy is that despite the fact that in our 1-vector system the same promoter and terminator sequences were used to facilitate expression of all genes, loss of vector parts or loss of sIgA expression upon plant transformation was never observed. We therefore assume that our multi-cassette expression vectors are stable and recombination did not occur. These data suggest that the use of a multi-cassette vector is the most efficient strategy for transient expression of heteromultimeric protein complexes.

### Co-Expression of Alpha Heavy Chain and Kappa Chain Stabilizes Both Proteins

**FIGURE 4:**
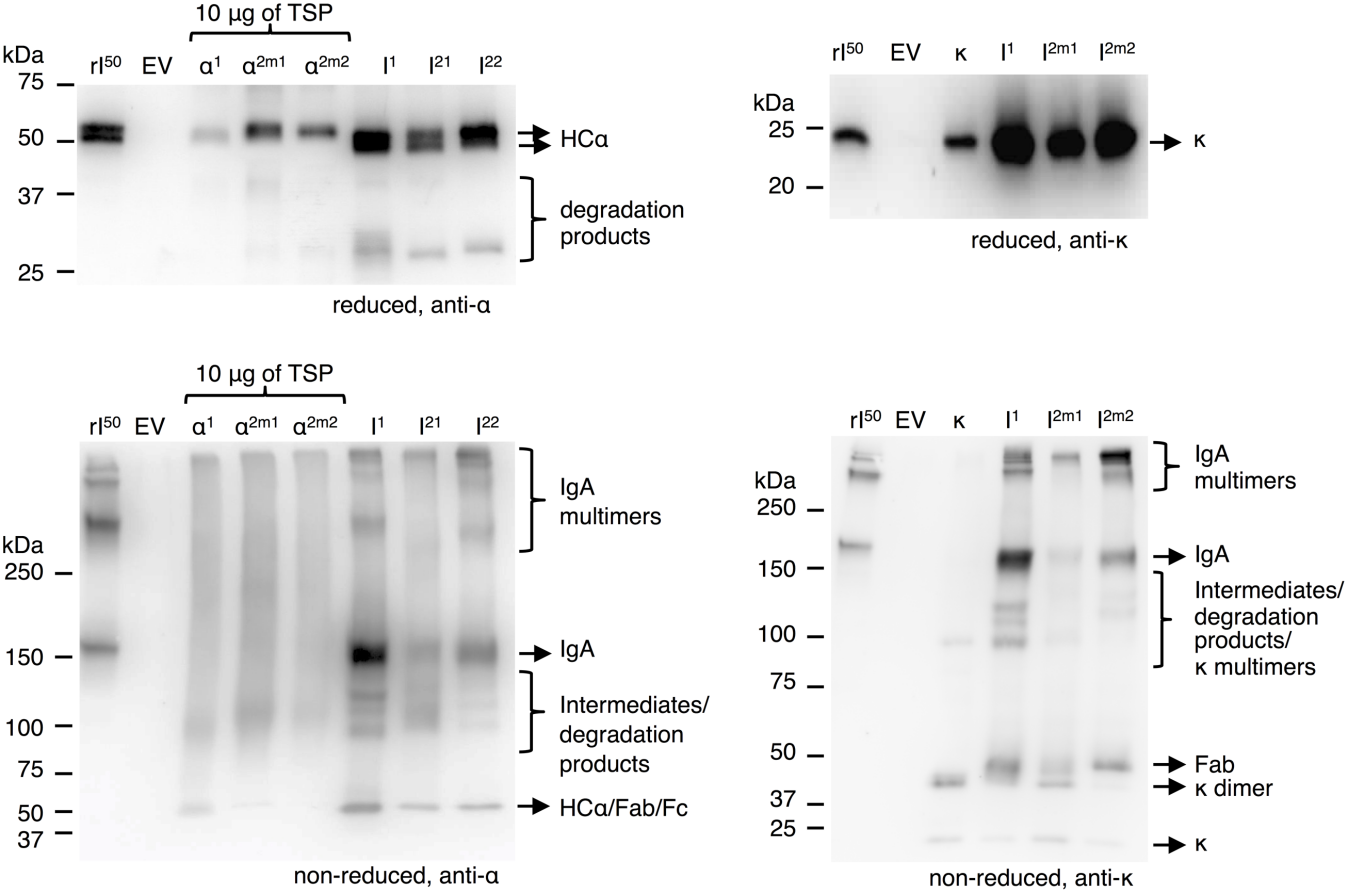
**IgA assembly using multi-cassette expression vectors**. Leaves were agro-infiltrated for the expression of alpha heavy chain 1 (α^1^), 2m1 (α^2m1^), 2m2 (α^2m2^), and kappa chain (κ) individually or co-expressed (I) using a multi-cassette vector and harvested 6 dpi. TSPs were extracted and 1 μg, unless stated otherwise, was separated by SDS-PAGE under reducing conditions followed by visualization using protein specific antibodies as indicated. As controls 50 ng of recombinant IgA1κ (rI^50^) and an EV sample were used.

To determine the limiting factor in sIgA assembly we first evaluated the efficiency of IgA assembly in the absence of the joining chain and secretory component. Thereto, we co-expressed the alpha heavy chains and the kappa chain using the dual-cassette expression vectors for all three IgA variants and compared it with the individual expression of the alpha heavy chains and kappa chains. Leaf extracts were analyzed by western blot under reducing and non-reducing conditions (**Figure 4**). Visualization of the alpha heavy chains and kappa chain under reducing conditions demonstrated that all proteins accumulate to a higher level upon co-expression. As the accumulation of the alpha heavy chains increases upon co-expression with the light chain the presence of a degradation product just above 25 kDa becomes clear. Considering the size of this degradation product it is most likely the result of cleavage in the hinge region. All three alpha heavy chains seem sensitive to proteolysis of the hinge region.

While analyzing co-expression of alpha heavy chains with kappa light chain under non-reducing conditions, a band around the expected size for IgA (∼150 kDa) was detected on blots either treated with anti-alpha heavy chain or anti-kappa chain specific antibodies. We therefore assumed that this band represents intact IgA complex. Next to the 150 kDa band, several bands migrating >250 kDa were detected. As these bands were also seen in the recombinant IgA control we assume they represent dimers/multimers of IgA. Also, several bands <150 kDa were detected, which may represent assembly intermediates, degradation products and/or individual polypeptide chains. Proteolytic cleavage in the hinge region results in Fc and Fab fragments of the same size as an intact alpha heavy chain (∼50 kDa). A band of 50 kDa is clearly detected on both alpha heavy chain and the kappa chain specific blots and therefore most likely represents Fab fragments. Two bands only detected on the kappa chain specific blot just below 25 and 50 kDa most likely represent un-associated monomeric and dimeric kappa chains. Assuming no un-associated alpha heavy chain is present, we conclude that accumulation of the alpha heavy chain is the limiting factor for IgA yield, despite the fact that the alpha heavy chains stabilize upon co-expression with the kappa chain.

### IgA Dimerization is the Limiting Step in sIgA Assembly

**FIGURE 5:**
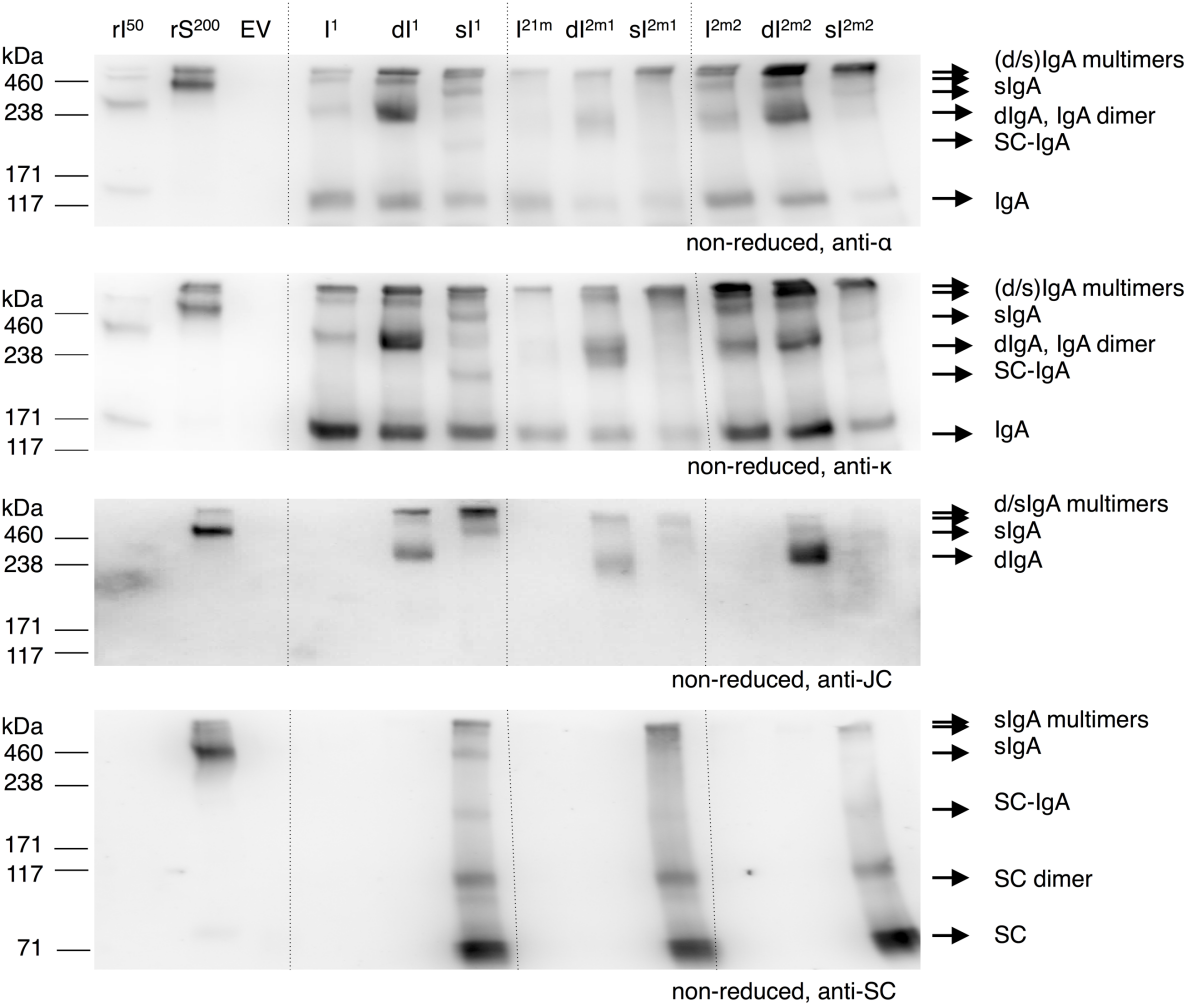
**sIgA assembly using multi-cassette expression vectors**. Leaves were agro-infiltrated for the expression of IgA1 (I^1^), 2m1 (I^2m1^), or 2m2 (I^2m2^) together with joining chain (dI) and secretory component (sI) using multi-cassette vectors and harvested 6 dpi. TSPs were extracted and 1 μg was separated by SDS-PAGE under reducing conditions followed by visualization using protein specific antibodies as indicated. As controls 50 ng of recombinant IgA1κ (rI^50^) and 200 ng purified sIgA (rS^200^) and an EV sample were used.

Next we evaluated the efficiency of sIgA assembly. We used the multi-cassette vectors to express IgA, dIgA, and sIgA (**Figure 3B**) with all three alpha heavy chain variants. Leaf extracts were analyzed on western blot under non-reducing conditions (**Figure 5**). Upon dIgA1, dIgA2m1, and dIgA2m2 expression two bands around 300 and >420 kDa were detected on the joining chain specific blot (third panel from the top). These bands can also be seen on the alpha heavy chain and kappa chain specific blots (first and second panel, respectively). We assume that these bands represent dIgA and multimerized (d)IgA. The 150 kDa band representing monomeric IgA in the alpha heavy chain and kappa chain specific blots is still present upon co-expression of the joining chain. This implies that dimerization of IgA is not 100% efficient or that the expression of the joining chain is limiting. The presence of free joining chains was not detected.

Upon sIgA1 expression monomeric IgA (∼150 kDa) is still detected on the alpha heavy chain and kappa chain specific blots. Next to that, bands of <100, ∼220, ∼400 and >420 kDa were detected on the secretory component specific blot (bottom panel). Because the band <100 kDa was only detected on the secretory component specific blot, this band most likely represents free secretory component. The 220 kDa band was also detected on the alpha heavy chain and kappa chain specific blot, but not on the joining chain specific blot and therefore most likely represents secretory component associated with monomeric IgA. Both the ∼400 and >420 kDa bands were also detected on the alpha heavy chain, kappa chain and joining chain blot and therefore must represent sIgA and multimers of (d/s)IgA. Upon sIgA2m1 and sIgA2m2 expression only the band <100 kDa representing free secretory component and the band >420 kDa representing multimeric sIgA was clearly distinguished. It may be that alpha heavy chains 2m1 and 2m2 are more inclined to multimerization. No dIgA was detected upon expression of all four genes using any of the alpha heavy chains. Apparently sIgA assembly is equally efficient for all three alpha heavy chains and the expression of the secretory component is not limiting. The latter can also be concluded by the ample presence of free secretory component in the sIgA samples.

**FIGURE 6:**
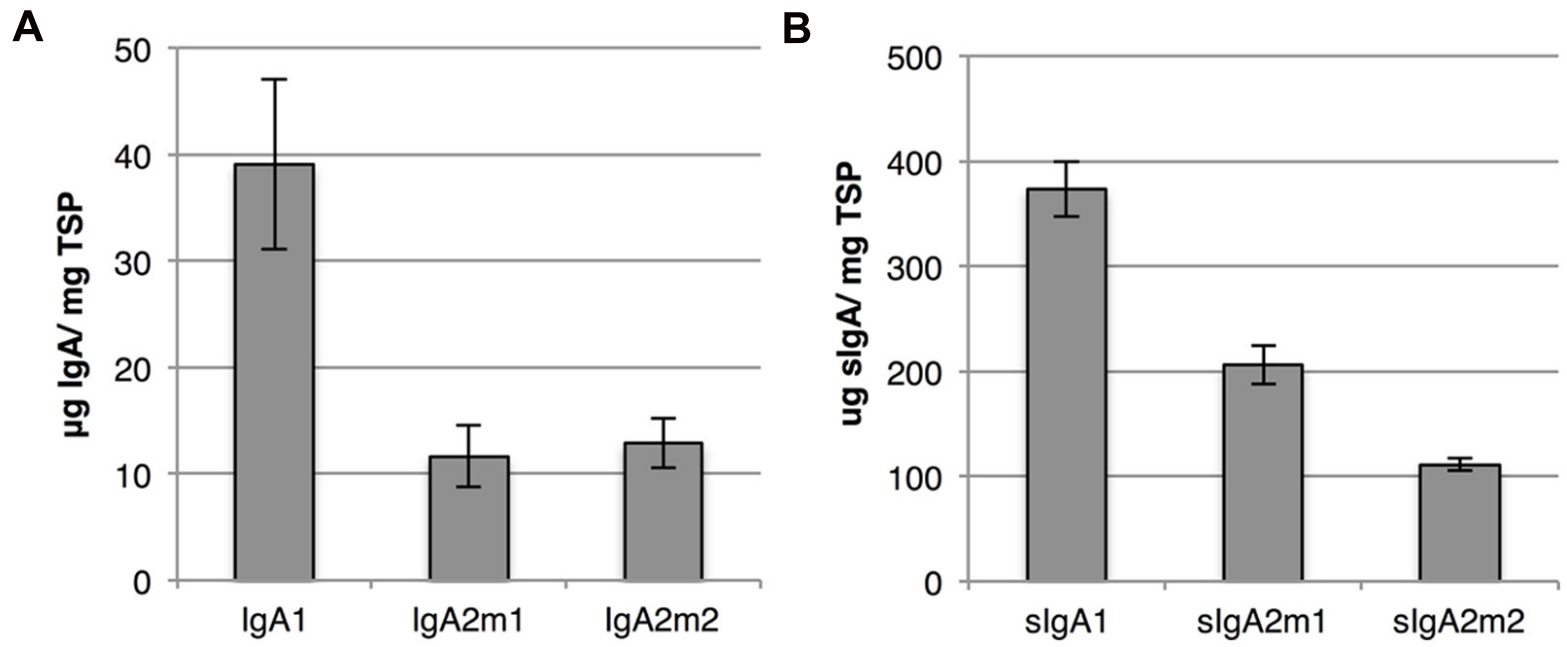
**IgA and sIgA yield using multi-cassette expression vectors. (A)** Average IgA yield of three biological replicates upon expression of IgA1, IgA2m1, and IgA2m2 using multi-cassette vectors harvested 6 dpi. IgA yield in leaf extracts was determined by sandwich ELISA using a kappa chain specific capture and alpha heavy chain specific detection antibody. Error bars indicate standard error. **(B)** Average sIgA yield of three biological replicates upon expression of sIgA1, sIgA2m1, and sIgA2m2 using multi-cassette vectors (vector system 1 in **Figure 3B**) and harvested 6 dpi. sIgA yield in leaf extracts was determined by sandwich ELISA using a kappa chain specific capture and secretory component specific detection antibody. Error bars indicate standard error.

Both IgA and sIgA yield were determined with a sandwich ELISA using an anti-light chain capture antibody and an anti-alpha chain or anti-secretory component detection antibody, respectively. Although we speak of IgA and sIgA yield, it should be stated that also IgA and sIgA intermediates may be detected. While yields of the IgA variants ranged between 10 and 40 μg/mg TSP, sIgA yield was at least ninefold higher for all variants (**Figure 6**). The higher sIgA yield, compared to IgA yield, may be explained by the fact that sIgA assembly prevents proteolytic degradation of IgA. However, sIgA yield may be somewhat overestimated due to the presence of monomeric IgA associated with the secretory component.

These western blotting and ELISA results show that dimerization of IgA is the limiting step for sIgA assembly. Improving dIgA assembly could further increase sIgA yield and reduce the presence of assembly intermediates thereby simplifying down-stream processing.

### Joining Chain Incorporation is the Limiting Factor for sIgA Yield

**FIGURE 7:**
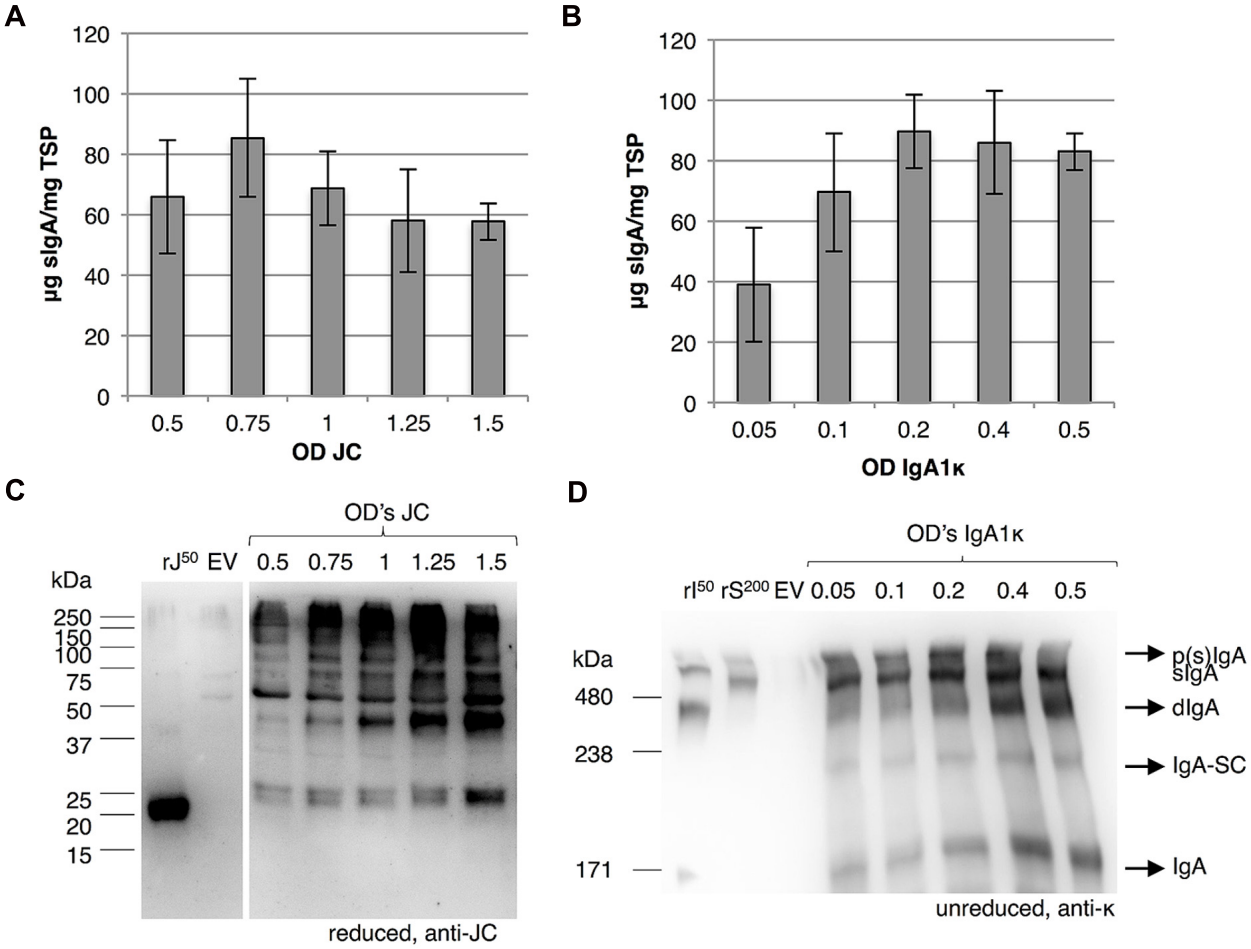
**Incorporation of the joining chain is the limiting factor for sIgA yield**. Leaves were agro-infiltrated for the expression of IgA1, JC and SC by co-infiltration (vector system 3 in **Figure 3B**) and harvested 6 dpi. **(A)** Average sIgA yield (*n* = 3, error bars indicate standard error) and **(B)** western blot analysis upon increasing the OD of the *Agrobacterium* culture facilitating joining chain expression. As controls recombinant joining chain (rJ^50^) and an EV sample were used. **(C)** Average sIgA yield of three biological replicates and **(D)** western blot analysis of sIgA assembly upon decreasing OD of the *Agrobacterium* culture facilitating IgA expression. As controls 50 ng of recombinant IgA1κ (rI^50^) and 200 ng purified sIgA (rS^200^) and an EV sample were used.

Next, we investigated whether joining chain expression is the limiting factor for sIgA assembly. Thereto, we attempted to increase the expression of the joining chain by increasing the OD of the *Agrobacterium* culture harboring the vector for joining chain expression using the 3-vector system. sIgA yield was determined by sandwich ELISA and joining chain expression was evaluated using western blot analysis (**Figures 7A,B**). While the expression of the joining chain increased by using a higher OD of the *Agrobacterium* culture harboring the vector for the joining chain expression, sIgA yield did not increase. We therefore assume that not joining chain expression, but its incorporation in the dIgA complex is the limiting factor for sIgA yield.

On a side note, the band that most likely represents monomeric joining chain (∼25 kDa) appears to be a doublet (two bands migrating very close to each other). A doublet may represent the same protein with a different number of *N*-glycans. Because the joining chain harbors only one *N*-glycosylation site, this doublet should represent non-glycosylated and glycosylated joining chain.

To evaluate if lowering IgA expression would influence the IgA:sIgA ratio, we also attempted to decrease the amount of IgA by reducing the OD of the *Agrobacterium* culture harboring the vector for IgA expression using the 3-vector system. Again sIgA yield was determined by sandwich ELISA and sIgA assembly was evaluated using western blot analysis (**Figures 7C,D**). Upon reduction of the OD of the *Agrobacterium* culture harboring the vector for IgA expression the amount of monomeric IgA reduces, but does not disappear. Also sIgA yield is reduced when the OD of the *Agrobacterium* culture that harbors the vector for IgA expression becomes lower than 0.2. Thus, the IgA:sIgA ratio does not change by lowering the expression of IgA. In other words, sIgA assembly cannot be improved by adjusting the expression of its individual components. It is therefore likely that not the capacity of the plant cell to assemble the protein complex, but intrinsic properties of the individual proteins determine dIgA assembly efficiency.

Moreover we also observed a significant proportion of dIgA when using ODs of 0.4 and 0.5 for IgA expression. In the previous results section we concluded that secretory component expression and association with dIgA was not limiting, as we hardly observed dIgA for any of the sIgA variants upon secretory component co-expression (**Figure 5**). However, the experiment described in the previous section was performed with the 1-vector system and the experiment described in this section was performed with the 3-vector system. This suggests that a significant proportion of cells does not receive the secretory component expression cassette upon co-infiltration or that at least the expression of the secretory component may vary from cell to cell.

## Discussion

We have studied the plant-based expression and assembly of three sIgA variants of the clinical antibody Ustekinumab (CNTO1275). We focussed on transient expression in *N. benthamiana*, as transient expression often yields more protein compared to stable transformation. Because sIgA is a heteromultimeric protein complex transient expression can be achieved in several ways. *Agrobacterium* cultures each harboring expression vectors that facilitate expression of the individual components can be co-infiltrated or a multi-cassette expression vector facilitating expression of the four components can be used. Also, a combination of these two strategies may be adopted. The risk with co-infiltration is that perhaps a proportion of the plant cells will not be transformed with all genes. This may result in the presence of un-associated components or assembly intermediates that may complicate downstream processing. Use of a multi-cassette vector would ensure that each transformed cell expresses each gene, however, the much larger T-DNA may be less efficiently transferred into the plant cells.

We co-infiltrated all genes individually (4-vector system), co-infiltrated the secretory component and joining chain with the multi-cassette vector for IgA1κ expression (3-vector system), co-infiltrated the secretory component and the multi-cassette vector for dIgA1κ expression (2-vector system) and finally also used a single multi-cassette vector for sIgA1κ expression (1-vector system). While sIgA yield was similar between the 4-, 3-, and 2-vector systems, surprisingly the use of the 1-vector system increased yield twofold. This yield increase may be explained by an increased number of cells that receive and express all genes. This hypothesis is supported by the fact that the presence of dimeric IgA was more dominant using the 3-vector system compared to the 1-vector system. This suggests that the secretory component is not expressed in all cells when using a separate vector for its expression. However, when transformation efficiency is a yield-limiting factor for sIgA, a yield increase would be expected every time the number of expression vectors is reduced. As mentioned, the 4-, 3-, and 2-vector systems yielded a similar amount of sIgA. Perhaps the T-DNA containing the gene for the secretory component is not efficiently transferred to plant cells, while all others reach most cells even when separate vectors are used. The T-DNA containing the secretory component gene is the largest of the four (joining chain 4.6 kbp, kappa chain 4.8 kbp, alpha heavy chain 5.6 kbp and the secretory component 5.9 kbp) and transformation efficiency has been demonstrated to go down with increased insert size (Frary and Hamilton, 2001). When the size of a T-DNA decreases transformation efficiency it would also reduce the efficiency by which the much larger T-DNA’s of the multi-cassette vectors are transferred into plant cells (IgA 7.7 kpb, dIgA 9.6 kpb and sIgA 12.8 kpb). This means that the yield increase due to the fact that more cells receive all genes every time the number of expression vectors is reduced, is compensated by a reduced number of transformed cells. Only in the case of the 1-vectory system the yield increase is higher than the yield loss due to lower transformation efficiency because each transformed cell expresses all genes.

Next to ensuring that all genes are transferred to each transformed plant cell, the 1-vector system has another benefit. The 1-vector system also enables an increased copy number of each expression cassette. With the 4-vector system each *Agrobacterium* culture was used with an OD of 0.5 giving the final infiltration mix an OD of 2.5 (also including an *Agrobacterium* culture for expression of the viral silencing inhibitor p19 with an OD of 0.5), which we consider a maximum OD to allow efficient infiltration. With the 1-vector system an OD of 2.0 can be used until this maximum is reached. This means that the presence of each expression cassette is increased fourfold. While this may not results in a fourfold increase of each expression cassette in the plant cells due to a lower transformation efficiency of larger T-DNAs, it did increase sIgA yield 1.6-fold.

While the 1-vector system facilitated the highest yield of our protein complex, co-infiltration may still yield more protein complex if the expression of the individual genes does not reflect the stoichiometric ratio of the protein complex. If so, the expression of individual genes may be adjusted by controlling the concentration of the *Agrobacterium* cultures to best reflect the stoichiometric ratio of the protein complex. If free polypeptides are found, it can never be concluded whether or not these arise from unbalanced expression or are a consequence of partial transformation. Alternatively, use of promoters with different strengths could be used that enable the accumulation of the individual proteins in the stoichiometric ratio of sIgA and allow the use of a 1-vector system. While testing of different promoters may be very laborious, systems are arising that allow easy high throughput cloning, such as the golden gate system (Engler et al., 2009).

Even when using a single multi-cassette expression vector, accumulation of assembly intermediates still occurred, with monomeric IgA as the most predominant intermediate. This is in line with four other studies on expression of murine, chicken and human sIgA in plants that all report the accumulation of a significant proportion of monomeric IgA next to sIgA (Ma et al., 1995; Wieland et al., 2006; Juarez et al., 2013; Paul et al., 2014). Follow up studies on the expression of murine (s)IgA demonstrated that this antibody was targeted to the vacuole due to a cryptic targeting signal in the tailpiece of murine IgA (Frigerio et al., 2000; Hadlington et al., 2003). In our previous publication on expression of monomeric human IgA we demonstrate that also human IgA is poorly secreted from plant cells (Westerhof et al., 2014). The tailpieces of both human and chicken IgA contain similar sequences as the suggested cryptic targeting signal of murine IgA. Thus, it may well be that also chicken and human IgA are targeted to the vacuole. If IgA is targeted to the vacuole, it is possible that a proportion of IgA is transported to the vacuole before the joining chain can be incorporated. Unfortunately the tailpiece of IgA cannot be removed, as the penultimate cysteine residue forms a disulphide bond with a free cysteine of the joining chain (Atkin et al., 1996). An investigation whether mutations in the cryptic vacuolar targeting signal can abolish vacuolar targeting without influencing the complex assembly may provide a solution.

Reports on the expression of sIgA in Chinese hamster ovary (CHO) cells also identify dIgA assembly as the yield-limiting step in sIgA expression (Berdoz et al., 1999; Li et al., 2014). Because mammalian cells are devoid of vacuoles, vacuolar targeting cannot explain the lack of sIgA assembly. Unfortunately, both studies did not determine joining chain expression. Thus it is unclear if sIgA assembly in CHO cells is caused by limited joining chain expression or inefficient incorporation of the joining chain. To evaluate if either joining chain expression or incorporation was the limiting step for the yield of our sIgA we increased the bacterial OD of the *Agrobacterium* culture. Increasing the *Agrobacterium* concentration facilitated increased joining chain expression. Even though joining chain expression was increased, sIgA yield was not, nor had the proportion of monomeric IgA diminished. We therefore assume that joining chain incorporation was the limiting step for sIgA assembly.

We also observed that the band assumed to represent monomeric joining chain migrated as a doublet. Because the joining chain harbors only one *N*-glycosylation site it is possible that this doublet represents a non-glycosylated and a glycosylated version of the joining chain. It was demonstrated that incorporation of the joining chain is reduced if asparagine 48 of the joining chain or asparagine 549 of the alpha heavy chain is not glycosylated (Atkin et al., 1996; Krugmann et al., 1997). In a previous study, we already confirmed that the *N*-glycosylation of the asparagine 549 of the alpha heavy chain is partial (Westerhof et al., 2014). Partial *N*-glycosylation as a reason for inefficient incorporation of the joining chain coincides with the fact that also reduced expression of IgA did not alter the IgA:sIgA ratio. We therefore hypothesize that the capacity of the plant cell to assemble dIgA is not limiting. Partial *N*-glycosylation of IgA and/or joining chain explains inefficient sIgA assembly both for plant as well as CHO cell produced sIgA. Because *N*-glycosylation is co-translational limited access to the *N*-glycosylation site cannot explain inefficient *N*-glycosylation. However, in a large-scale analysis of glycoproteins it was suggested that the sequence surrounding an *N*-glycosylation signal may influence *N*-glycosylation efficiency (Petrescu et al., 2004). Perhaps adaptation of the sequences surrounding the *N*-glycosylation sites in the joining chain and tailpiece of the alpha heavy chain can increase *N*-glycosylation efficiency. If this would increase sIgA assembly and reduce the presence of sIgA intermediates it would not only increase yield, but also simplify purification procedures.

Taken together our data suggests that plants most certainly allow the economic production of heteromultimeric protein complexes such as sIgA. The maximum yield of our sIgA1κ-Ustekinumab variant was 37% of TSP, which is well above the 1% commercial viability threshold. Hereby transient expression with use of a multi-cassette expression vector is the best strategy, because it ensures expression of all genes in all transformed cells. This prevents the occurrence of assembly intermediates due to partial transformation.

## Author Contributions

LW – Has had the lead in this research project (concept and design), acquisition of data, analysis and interpretation of data, drafting and revising the article, and final approval of the version to be published.

RW – Substantial contributions to concept and design, acquisition of data, analysis and interpretation of data, and writing and revising the article critically for important intellectual content and final approval of the version to be published.

DvR – Acquisition of data.

CvW – Acquisition of data.

AG – Writing and revising the article critically for important intellectual content.

JB – Writing and revising the article critically for important intellectual content.

AS – Writing and revising the article critically for important intellectual content and final approval of the version to be published.

## Conflict of Interest Statement

The authors declare that the research was conducted in the absence of any commercial or financial relationships that could be construed as a potential conflict of interest. The reviewer Marcello Donini and handling Editor Eugenio Benvenuto declared their shared affiliation, and the handling Editor states that, nevertheless, the process met the standards of a fair and objective review.
